# Non-Islet Cell Tumor Hypoglycemia Caused by Recurrent Pelvic Solitary Fibrous Tumor

**DOI:** 10.7759/cureus.12878

**Published:** 2021-01-23

**Authors:** Diana Pinho dos Santos, Rui Correia, Adelino Carragoso, Carlos Casimiro, Ana Lemos

**Affiliations:** 1 Department of Internal Medicine, Centro Hospitalar Tondela-Viseu, Epe, Viseu, PRT; 2 Department of General Surgery, Centro Hospitalar Tondela-Viseu, Epe, Viseu, PRT

**Keywords:** hypoglycemia, non-islet cell tumor hypoglycemia, solitary fibrous tumor, doege-potter syndrome

## Abstract

Non-islet cell tumor hypoglycemia is a rare paraneoplastic condition caused by an extra-pancreatic tumor. We report a rare case of hypoglycemia caused by a relapsing pelvic solitary fibrous tumor associated with Big-IGF-2 production.

A 72-year-old woman was admitted to our hospital because of loss of consciousness and hypoglycemia. She had a history of ovarian solitary fibrous tumor, which has relapsed. From investigation, serum levels of insulin and C-peptide were suppressed; IGF-1 was slightly reduced and IGF-2 was within the normal range, but the IGF-2: IGF-1 ratio was elevated, indicating the presence of Big-IGF-2 secreting non-islet cell tumor. Contrast-enhanced computed tomography (CT) showed a large pelvic mass. She was then submitted to surgical resection of the mass, which histologically proved to be a solitary fibrous tumor. Three months later, she remains asymptomatic.

Non-islet cell tumor hypoglycemia should be considered in the differential diagnosis of patients presenting with tumors and recurrent hypoglycemia.

## Introduction

Non-islet cell tumor hypoglycemia (NICTH) is a rare cause of refractory hypoglycemia that is associated with large tumors usually of epithelial or mesenchymal origin such as solitary fibrous tumor (SFT) [1-2]. In most cases, it is caused by tumoral secretion of incompletely processed insulin-like growth factor (IGF)-2, termed Big-IGF-2 or pro-IGF-2 [3-4].

The majority of SFTs are thoracic [5]. They can arise at any age but they are more common between the fifties and sixties [5]. The incidence is similar in both genders [5]. Usually, SFTs have a benign clinical course, but the tumor may recur and metastasize after surgical resection [2]. Approximately 10% to 20% are categorized as malignant [2,6].

We report a case of hypoglycemia caused by a relapsing pelvic SFT associated with Big-IGF-2 production. The patient underwent tumor resection and immediately normalized her glycemia.

## Case presentation

A 72-year-old Caucasian woman was admitted to the emergency department with loss of consciousness. It was documented hypoglycemia (capillary blood glucose of 30 mg/dL). In the previous two months, she had experienced repeated episodes of loss of consciousness and symptoms of malaise, confusion, tremors, and diaphoresis following prolonged fasting periods that resolved after eating. There were no noticed weight changes.

Her clinical history included essential hypertension and surgical resection of a benign SFT from the left ovary (fibrothecoma) approximately 11 years before. About a year ago, a follow-up contrast-enhanced CT showed a heterogeneous 10x6.9 cm pelvic mass, which has been under surveillance. Her medications included perindopril, amlodipine, indapamide, and furosemide. There is no history of the inappropriate use of insulin, oral antidiabetics, or steroids. She did not have a history of diabetes mellitus or bariatric surgery. She was a non-smoker and non-alcoholic. There was no evidence of diabetes in the family. Her physical examination was unremarkable. The patient was admitted for monitoring and further investigation.

In the ward, she continued to experience episodes of spontaneous hypoglycemia, regardless of meals, requiring an infusion of dextrose 10%. Laboratory workup revealed normal kidney and liver function. Hypokalemia of 2.8 mEq/L (normal value: 3.5-4.5) was observed. Thyroid function tests were normal. Fasting cortisol was 14.7 ug/dL (normal value: 5.27-22.45) with adrenocorticotropic hormone (ACTH) 22.0 pg/mL (normal value: 4.7-48.8). Considering their history of neoplasia, we extended the study. During a further hypoglycemic episode of 27 mg/dL, serum levels of insulin, C-peptide, and beta-hydroxybutyrate were suppressed; the concentration of IGF-1 was slightly reduced (45 ug/L; normal value: 60-215) and IGF-2 was within the normal range (626 ug/L; normal value: 210-750), but her insulin-like growth factor-binding protein-2 (IGFBP-2) was increased (1899 ng/mL; normal range: 110-744) and the IGF-2: IGF-1 ratio was 13.9 (normal value <3.0), indicating the presence of Big-IGF-2 secreting non-islet cell tumor (Table 1).

**Table 1:** Summary of laboratory results AST: aspartate aminotransferase; ALT: alanine transaminase; ACTH: adrenocorticotropic hormone; IGF: insulin-like growth factor

Test	Values	Reference range
Glucose (mg/dL)	27	74-106
Potassium (mmol/L)	2.8	3.5-4.5
AST (UI/L)	29	3-31
ALT (UI/L)	27	3-31
Creatinine (mg/dL)	0.5	0.5-1.2
Cortisol (ug/dL)	14.7	5.27-22.45
ACTH (pg/mL)	22.0	4.7-48.8
Insulin (uU/mL)	0.5	2.60-37.60
Proinsulin (pmol/L)	0.5	<5.1
C-peptide (ng/dL)	0.09	0.81-3.85
beta-hydroxybutyrate (mmol/L)	0.07	0.03-0.30
IGF-1 (ug/L)	45	60-215
IGF-2 (ng/mL)	626	210-750
IGF-2: IGF-1 ratio	13.9	<10.0

**Figure 1:**
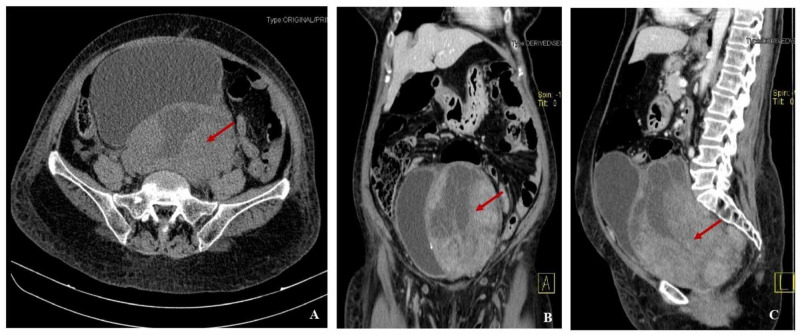
Contrast-enhanced CT image showing a pelvic large mass, measuring 17 cm of greater diameter, heterogeneous, well-defined, with cystic areas, deviating the bladder (arrows) (A) Axial section; (B) Coronal section; (C) Sagittal section CT: computed tomography

Thorax, abdomen, and pelvis contrast-enhanced CT demonstrated a large pelvic mass, measuring 17 cm, with greater diameter, heterogeneous, well-defined, with cystic areas, deviating the bladder, probably corresponding to an ovarian tumor; no secondary lesions or lymphadenopathy were described (Figure 1).

**Figure 2:**
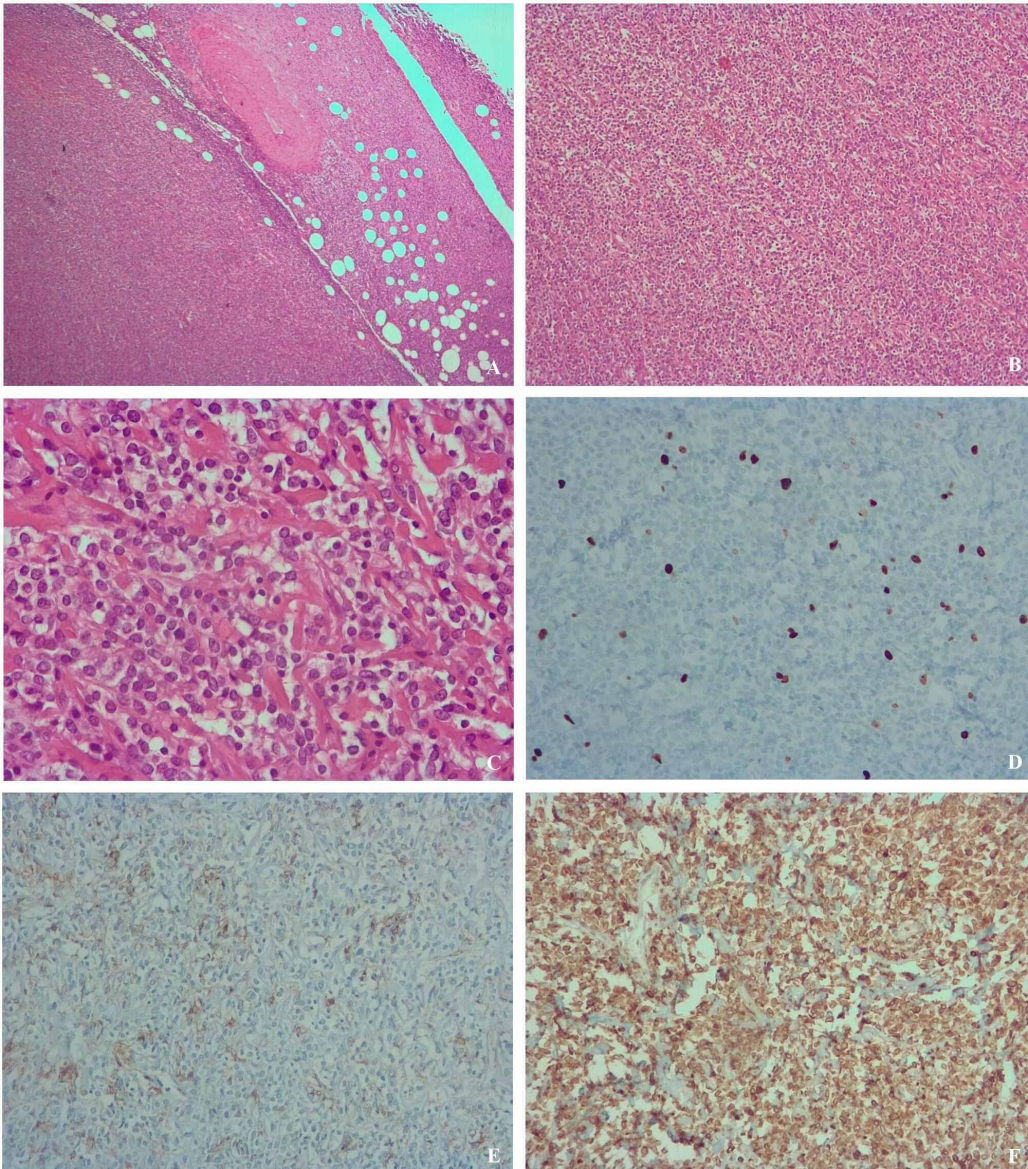
Microscopic examinations (A) Well-defined lesion with apparent peripheral adipose tissue (hematoxylin and eosin stain 40x); (B) and (C) Spindle cells densely arranged on a collagen background, with oval nuclei, eosinophilic cytoplasm, indistinct boundaries, and no definite pattern (C – hematoxylin and eosin stain 100x and D - hematoxylin and eosin stain 400x); (D) Immunohistochemical stain for Ki-67 <10% (200x); (E) Immunohistochemical stain for CD34 shows focal positivity (200x); (F) Immunohistochemical stain for BCL2 shows diffuse positivity (200x)

The patient was then submitted to surgical resection of the tumor, which histologically proved to be mesenchymal neoplasia consisting of spindle cells, without evident pleomorphism; no significant mitotic activity or necrosis was identified; immunohistochemical staining showed diffuse positivity for BCL2 and focal positivity for CD34, with negativity for CD99, S100, desmin, caldesmon, and alpha-actin; the proliferative index for Ki-67 was less than 10%. These features were consistent with the relapse of ovarian SFT (Figure 2).

Hypoglycemic episodes resolved immediately after surgery. The patient was discharged one month after the initial admission and remained completely asymptomatic three months later. No further hypoglycemic episodes were documented.

## Discussion

Spontaneous and recurrent hypoglycemia in the absence of hypoglycemic therapy is a medical challenge because it can have multiple etiologies, namely, postprandial hypoglycemia, insulinomas, insulin autoimmune syndrome, adrenal insufficiency, and critical illness (for example, renal or liver failure) [7]. Uncommonly, it can be a result of paraneoplastic syndrome secondary to extra-pancreatic tumors being this association termed as NICTH and first reported in 1929 by Nadler et al. in a patient with hepatocellular carcinoma [8-9].

NICTH is associated with a variety of malignant and non-malignant tumors, usually of epithelial or mesenchymal origin [1-2,8], such as mesothelioma [8], hemangiopericytoma [8], adrenocortical carcinoma [8], pancreatic carcinoma [8], medullary thyroid carcinoma [8], lymphoma/leukemia [8], carcinoid syndrome [8], fibrosarcoma [7,8,10], gastrointestinal stromal tumor, renal cell carcinoma [7,10], prostate cancer, breast cancer, bladder cancer, and SFT [10]. Regarding SFT, NICTH is found in approximately 4% of cases [3,11] and was reported for the first time in literature, in 1930, by Doege and Potter [11-12].

SFT may be found in various anatomic sites [5,12], with 80% of them being located in the thoracic cavity. Among extra-thoracic STF, 16% were reported in the pelvic cavity [5].

According to Wada et al., there is approximately 10 cases of pelvic SFT with hypoglycemia described in the literature [5]. As mentioned previously, the majority of SFT has a benign clinical course, but about 10% to 20% show malignant behavior [2,6]. They also tend to recur more than 10 years after the initial diagnosis and metastasize post-surgical resection, regardless of whether the original tumors are benign or malignant [2].

The definitive diagnosis of SFT is based on histologic identification of spindle cell tumor with a distinctive fibroblastic morphology, comprising a patternless pattern of spindle cells, arranged as areas of alternating cellularity and hypocellular collagenous stroma, as well as CD34 (most cases), CD99, and/or BCL-2 expression [13-14]. The criteria for malignancy in SFT are increased cellularity, nuclear atypia, infiltrative growth, and mitotic count of more than four per 10 high-power fields [13]. Ki-67, a marker of cell proliferation, can be used to stratify lesions, and a rate higher than 12% is suggested as a cut-off level for malignancy [15-16]. In the case reported, Ki-67 <10% was verified, which indicates less aggressive behavior. The postoperative risk of recurrence or metastasis is seen to increase with a tumor size of >10 cm or if a malignant component is detected [13], therefore, considering the size of our patient's lesion, there is a high probability of relapse.

Hypoglycemia can be the presenting form of this kind of tumor, or it can appear after its diagnosis [8]. Our patient had a previous history of benign ovarian tumor resected more than 10 years ago; she had presented a pelvic mass about one year before symptoms of hypoglycemia, which was compatible with disease relapse. The tumor histology was classified as benign due to low mitosis and the absence of tumoral necrosis.

The most common cause of this type of hypoglycemia is the tumoral secretion of incompletely processed IGF-2, also known as Big-IGF-2 or pro-IGF-2 [3-4,9]. In 1988, Daughaday et al. first described high-molecular-weight IGF-2 in patients with NICTH [2,8-9]. Usually, mature IGF-2 forms a large ternary complex with IGF-binding protein (IGFBP) and with an acid-labile subunit. However, in NICTH, this ternary complex cannot be formed because of the excessive molecular weight of Big-IGF-2. Therefore, the levels of free Big-IGF-2 and binary complex increase, diffuse through the capillary wall, and bind to insulin receptors, resulting in insulin-like effects [9,17]. Big-IGF-2 can produce hypoglycemia in the setting of a normal IGF-2 level [8,10]. Even though Big-IGF-2 has glucose-lowering potency that is approximately 5% of insulin’s potency, its concentration in serum is 1000 times greater [10]. Similar to insulin, Big-IGF-2 inhibits hepatic gluconeogenesis, glycogenolysis, ketogenesis, and lipolysis, and it increases the uptake of glucose by muscles [8]. In addition, it may lead to reduced growth hormone secretion [9] and IGF-1 levels via the activation of IGF-1 receptors in the hypothalamus by IGF-2 [8]. Glucagon production is also suppressed due to the activation of IGF-1 receptors on the pancreatic alpha cells by IGF-2 [8]. Glucose consumption by the tumors does not appear to contribute significantly to hypoglycemia [8].

Diagnosis of hypoglycemia is supported by Whipple’s triad (low plasma glucose, symptoms and/or signs of hypoglycemia, and resolution after hypoglycemia correction) [7]. Our patient presented with a history of two months of neuroglycopenic (confusion, loss of consciousness) and autonomic manifestations (tremor, sweating) [7]. However, life-threatening clinical events, such as seizures and coma, can be presentation forms of hypoglycemia [7].

She had symptoms in a fasting state, which automatically allows us to exclude postprandial hypoglycemia (reactive hypoglycemia); besides that, our patient had no history of gastrointestinal surgery (for example, bariatric surgery), so excluding dumping syndrome that is an example of postprandial hypoglycemia.

Although our patient had no acromegaloid changes, they have been described in rare instances secondary to the activation of IGF receptors by IGF-2 [8]. In the evaluation of hypoglycemia, it is important to get a detailed history, to evaluate any systemic diseases or medications that can predispose to hypoglycemia [8]. Our patient had no history of diabetes mellitus or use of hypoglycemic agents, which allow us to exclude induced hypoglycemia. Diagnostic workup includes also the measurement of insulin, proinsulin, C-peptide, and beta-hydroxybutyrate level. Other laboratory tests that are important to request include IGF-1, IGF-2, and growth hormone (GH) levels. Unfortunately, assays for Big-IGF-2 are not commercially available [8,10].

NICTH presents with low levels of insulin, C-peptide, proinsulin, and beta-hydroxybutyrate [8,10]. IGF-1 is typically suppressed while the levels of IGF-2 may be normal or elevated [2,8,10]. The molar ratio of IGF-2/IGF-1 > 3 (often approaching 10 or more) [7,8,10] is diagnostic of NICTH [2,7,10].

Plasma insulin, C-peptide, and proinsulin values are elevated in patients with insulinoma, hypoglycemic agent-induced hypoglycemia, and insulin autoimmune syndrome, so in our case, we can exclude these etiologies. In the present case, IGF-1 levels were suppressed in the serum, effectively ruling out an IGF-1 producing tumor [9]. Concurrent normal morning cortisol rules out adrenal insufficiency. Renal and liver dysfunction associated with a tumor, which could contribute to hypoglycemia, were not present in our patient [8,10]. The mechanism that causes hypokalemia remains unclear, however, it has been postulated to be secondary to the insulin-like activity of IGF-2 [17].

Surgical excision of the tumor is the most definitive treatment of NICTH [2,8,10]. It results in an immediate resolution of hypoglycemia [10], as seen in our case. Sometimes, complete resection of the tumor may not be viable. In these cases, frequent meals and/or intravenous dextrose infusions [9] are used to control hypoglycemic episodes and even parenteral nutrition may be necessary. Chemotherapy and radiotherapy to reduce the tumor burden may help symptom relief. Glucagon [8-9], diazoxide, octreotide, glucocorticoids [2,8-9], and growth hormone [8-9] (either alone or in varying combinations) are also possible used in selected cases, although the overall prognosis of the condition is poor [7]. Metastatic disease and tumor recurrence are associated with a poor prognosis [10].

As NICTH results from the interaction of Big-IGF-2 and the insulin receptor, therapies aimed at disrupting this interaction have been developed. Antibodies to IGF-2 and a prohormone convertase that converts pro to mature IGF-2 are being studied [8].

## Conclusions

This case report has described a rare case of NICTH associated with a relapsed pelvic SFT. It is important to recognize that unexplained hypoglycemia, in patients with no history of diabetes or weight loss surgery, can be a paraneoplastic phenomenon. Currently, there is limited knowledge regarding this rare paraneoplastic syndrome and, in cases where surgical resection is not possible, new therapies need to be developed.
